# Clinical-pathological features and muscle imaging findings in 36 Chinese patients with rimmed vacuolar myopathies: case series study and review of literature

**DOI:** 10.3389/fneur.2023.1152738

**Published:** 2023-04-28

**Authors:** Xiao-jing Wei, Hui Sun, Jing Miao, Ru-qing Qiu, Zong-zhi Jiang, Zhen-wei Ma, Wei Sun, Xue-fan Yu

**Affiliations:** Department of Neurology and Neuroscience Center, The First Affiliated Hospital of Jilin University, Changchun, Jilin, China

**Keywords:** vacuolar myopathy, differential diagnosis, muscle magnetic resonance imaging, autophagy, next generation sequencing

## Abstract

**Introduction:**

Rimmed vacuolar myopathies (RVMs) are a group of genetically heterogeneous diseases that share histopathological characteristics on muscle biopsy, including the aberrant accumulation of autophagic vacuoles. However, the presence of non-coding sequences and structural mutations, some of which remain undetectable, confound the identification of pathogenic mutations responsible for RVMs. Therefore, we assessed the clinical profiles and muscle magnetic resonance imaging (MRI) changes in 36 Chinese patients with RVMs, emphasizing the role of muscle MRI in disease identification and differential diagnosis to propose a comprehensive literature-based imaging pattern to facilitate improved diagnostic workup.

**Methods:**

All patients presented with rimmed vacuoles with varying degrees of muscular dystrophic changes and underwent a comprehensive evaluation using clinical, morphological, muscle MRI and molecular genetic analysis. We assessed muscle changes in the Chinese RVMs and provided an overview of the RVMs, focusing on the patterns of muscle involvement on MRI.

**Results:**

A total of 36 patients, including 24 with confirmed distal myopathy and 12 with limb-girdle phenotype, had autophagic vacuoles with RVMs. Hierarchical clustering of patients according to the predominant effect of the distal or proximal lower limbs revealed that most patients with RVMs could be distinguished. GNE myopathy was the most prevalent form of RVMs observed in this study. Moreover, MRI helped identify the causative genes in some diseases (e.g., desminopathy and hereditary myopathy with early respiratory failure) and confirmed the pathogenicity of a novel mutation (e.g., adult-onset proximal rimmed vacuolar titinopathy) detected using next-generation sequencing.

**Discussion:**

Collectively, our findings expand our knowledge of the genetic spectrum of RVMs in China and suggest that muscle imaging should be an integral part of assisting genetic testing and avoiding misdiagnosis in the diagnostic workup of RVM.

## 1. Introduction

Rimmed vacuolar myopathies (RVMs) are an emerging group of etiologically diverse muscle disorders characterized by dysregulated autophagy accompanied by rimmed vacuoles (RV) in the skeletal muscle (1). These RVs exhibit cleared-out spaces surrounded by a rim of basophilic granular material in the fiber sarcoplasm.

The clinical phenotypes of RVM cover a wide and overlapping clinical spectrum. Therefore, the confirmatory diagnosis of RVM tends to rely on next-generation sequencing (NGS) rather than on clinical characteristics alone. Currently, RVM is seldom considered in the differential diagnosis by neuroradiologists; thus, diagnosis remains challenging. The current literature regarding the imaging of RVM is limited to sporadic case reports and case series. In 2020, Mair et al. reported the differential diagnosis of vacuolar myopathies in the NGS era based on clinical and pathological features (1). This approach theoretically facilitates differential diagnosis; however, identifying the pathogenic mutations responsible for RVM is complicated by the presence of non-coding sequences and structural mutations, some of which remain undetectable. Therefore, it is necessary to support a differential diagnosis by establishing better tests for different RVMs.

Muscle magnetic resonance imaging (MRI) has become a powerful tool for diagnosing muscle disorders (2, 3). It helps to establish an accurate diagnosis based on the recognition of distinctive patterns of affected muscles and thus guides molecular genetic testing. For instance, the trefoil with single fruit sign (4) and concentric fatty infiltration pattern (5) is highly specific for dystrophinopathies and limb-girdle muscular dystrophy R9 (LGMD R9, previously known as LGMD2I), respectively. Furthermore, there have been no related studies on alterations in muscle MRI in RVM. Therefore, we aimed to discuss the genotypic spectrum and muscle imaging abnormalities in RVM and proposed a comprehensive literature-based imaging pattern that can help in the diagnostic workup.

## 2. Materials and methods

### 2.1. Population

A total of 27 index patients and nine additional family members who had presented at the Department of Neurology, Jilin University (Jilin, China) between 2001 and 2022 and exhibited a lesion of RVM were included in the study. A neurologist with a neuromuscular specialization performed a detailed neurological examination on several occasions. Patients with no definite pathogenic mutation or histologically proven inclusion body myositis were excluded. Genetic and clinical findings and biopsy results have already been published for two patients: P16 (6) and P26 (7).

### 2.2. Muscle biopsy

Open muscle biopsies were performed for diagnostic purposes from mildly to moderately affected skeletal muscles according to the clinical manifestations and MRI features. For further observations, unfixed sections were stained using standard histological, enzyme histochemical, and immunohistochemical techniques.

### 2.3. Muscle MRI

The MRI images were obtained using a 1.5T *MR scanner* (*MAGNETOM Avanto, Siemens*, Germany) and were analyzed to evaluate the general pattern of muscle involvement. Scans were performed at the thigh and calf levels. At both scan levels, every muscle involved was assessed on each side.

All 18 muscles were analyzed in each patient, containing the anterior thigh compartment (rectus femoris (RF), vastus medialis (VM), vastus intermedius (VI), and vastus lateralis (VL)) and the posterior thigh compartment [adductor longus (AL), adductor magnus(AM), gracilis(G), sartorius(S), semitendinosus(ST), semimembranosus (SM), and biceps femoris (BF)]; and the anterior leg compartment [tibialis anterior (TA), extensor digitorum longus (EDL), and peroneus longus (PL)] and the posterior leg compartment [tibialis posterior (TP), soleus (SOL), gastrocnemius medial (GCM), and gastrocnemius lateral (GCL)].

### 2.4. Genetic testing

Gene sequencing was performed mainly at the Beijing Kangso Medical Inspection, China. Genomic DNA was isolated from the peripheral blood samples of the trios using a DNA Midi Kit (Qiagen GmbH, Hilden, Germany). Custom-design in-solution hybridization (Agilent SureSelect, Agilent) was used to select panel genes. All known protein-encoding regions were captured and enriched. Whole-exome sequencing (WES) was performed on the Illumina HiSeq2500 and HiSeq4000 systems to an average read depth 100× covering 99.9% per sample. For WES enrichment, the Nextera Rapid Capture Exome (v1.2, Illumina, San Diego, CA, USA) was used. HiSeq reads were mapped to the human hg19 reference genome using the Burrows–Wheeler Aligner software. The Genome Analysis Toolkit (GATK) was used to detect duplicated reads and single-nucleotide variants. Patients and their relatives with non-conclusive genetic workup were followed up for further examination. Data were processed preliminarily according to the standard procedures of WES (8). Segregation analysis of the variants was performed in the available family members using Sanger sequencing. Repeat-primed PCR (RP-PCR) and GC-rich PCR (GC-PCR) were used for detecting CGG repeat expansions in *GIPC1*.

## 3. Results

### 3.1. General clinical and genetic findings

Comparative analysis of the clinical characteristics in the patients including myocardial and respiratory involvement and genetic results are shown in Supplementary Tables 1, 2. MRI imaging of the legs revealed focal fatty degeneration in 18 participants, diffuse infiltration in four patients, and widespread lipomatous alterations of all lower limbs' muscles in five patients. Panel NGS established variations in 15 patients in *GNE* (GNE myopathy) and *DYSF* (Dysferlinopathy), respectively. RP-PCR detected >70 CGG repeat expansions in *GIPC1*, and GC-PCR confirmed the number of repeats was 135. Whole-exome sequencing (WES) indicated one novel homozygous exon2 deletion in *TRIM32*, which was determined at the homozygous state with quantitative real-time polymerase chain reaction.

### 3.2. General histopathological findings

A total of 11 patients exhibited numerous (>10) vacuolar defects and 16 biopsies with light (1–3) to moderate (3–10) RVs were revealed in the samples.

### 3.3. RVM of distal muscles weakness with identified pathogenic mutation

#### 3.3.1. GNE myopathy

**Figure 1:**
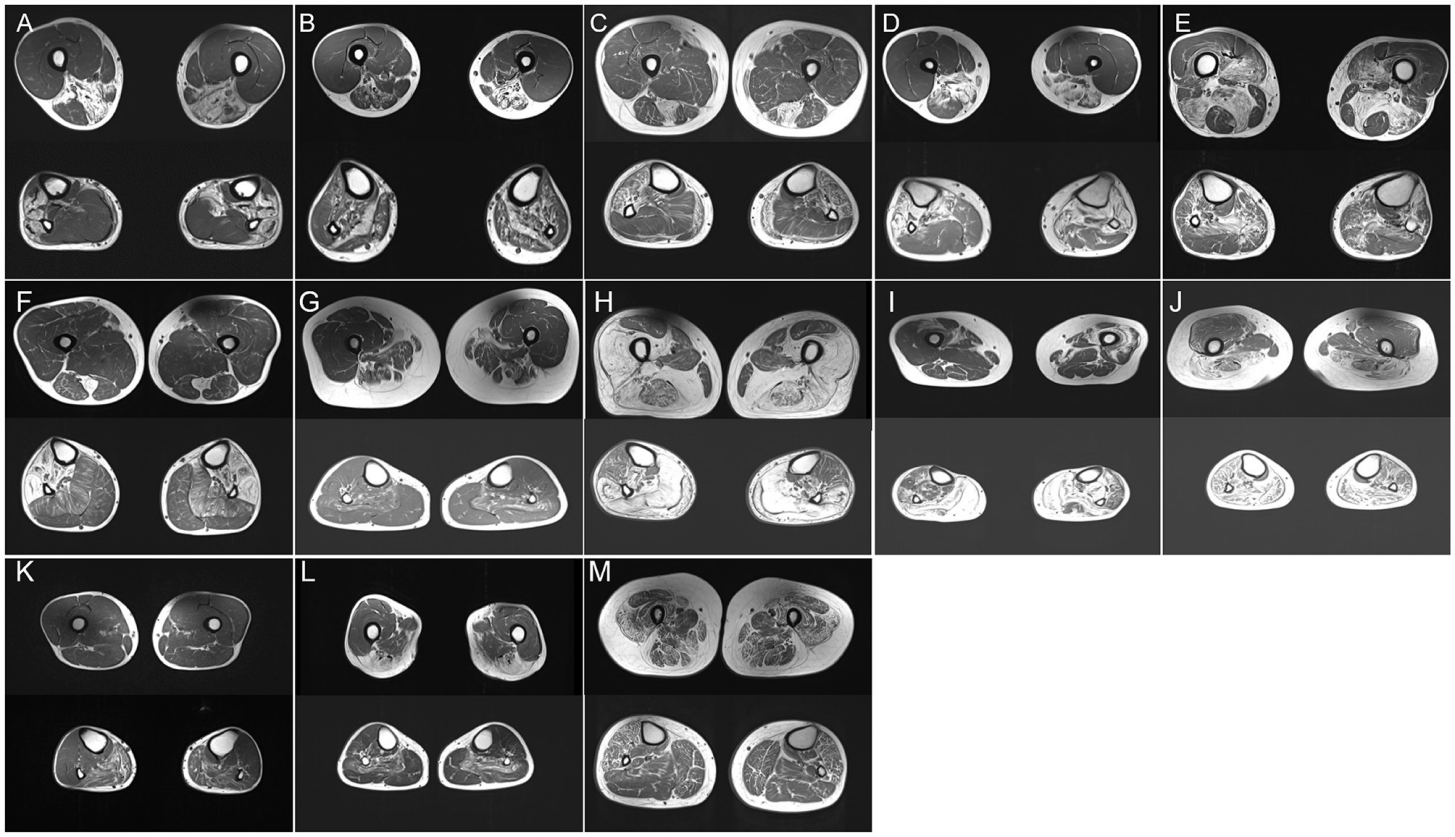
**(A–M)** Thigh and calf muscle MRI (T1-weighted images) of P2 **(A)**, P12 **(B)**, P14 **(C)**, P15 **(D)**, P17 I, P18 **(F)**, P19 **(G)**, P20 **(H)**, P21 **(I)**, P22 **(J)**, P23 **(K)**, P24 **(L)**, and P27 **(M)**.

A total of 12 patients were diagnosed with GNE myopathy. All individuals primarily presented with weakness of the lower extremities, and all but five had proximal regions of muscle involvement. Muscle biopsies showed clusters of small angulated fibers with rimmed vacuoles (Supplementary Figures 1A, B). Lower-limb MRI documented that fatty changes were restricted to the biceps femoris, semimembranosus, semitendinosus, adductor magnus and gracilis muscles, and the tibialis anterior, extensor digitorum longus, and peroneus longus muscles in the distal leg (Figure 1A). Together with our previous studies (9, 10), genetic analysis of these individuals identified five novel mutations (c.455_456insC, c.1262C>T, c.859G>A, c.1426A>G, and exon1 deletion) and six previously reported mutations (c.131G>C, c.620A>T, c.653A>G, c.1726G>C, c.1807G>C, and c.2005G>C) in *GNE*. Exon 1 deletion was confirmed in P5 using real-time PCR analysis.

#### 3.3.2. Dysferlinopathy

A total of three patients (P11, P12, and P13), one from a Chinese intermarriage family (P11), experienced insidious progressive weakness of the limbs, distal-proximal limbs. Under a light microscope, classic subsarcolemmal autophagic vacuoles rounded with basophilic stippling were illustrated in all patients (Supplementary Figure 1C). Low immunoreactivity *to* dysferlin was seen in frozen sections. Fat replacement with a combination of calves (gastrocnemius medialis and soleus) and thighs (adductor major, semimembranosus, and semitendinosus) was observed on the MRI (Figure 1B). The distal distribution of muscle weakness and MRI patterns consistent with those of dysferlinopathy, targeted sequencing of *DYSF* revealed the mutation c.1471dupA, p. (Met491AsnfsTer15) in P11 in a homozygous state. P12 carried compound heterozygous mutations in *DYSF*: two frameshift mutations, c.3866delG, p. (Trp1289CysfsTer4) and c.4106delT, p. (Leu1369ArgfsTer8). P13 harbored compound heterozygous mutations, including a missense mutation c.2875 C>T, p. (Arg 959Trp) and a premature termination mutation c.4321C>T, p. (Gln1441X).

#### 3.3.3. Myofibrillar myopathy

##### 3.3.3.1. Desminopathy

P14 experienced fatigue and weakness in the lower limbs since the age of 35 years. Histopathological examination showed branching granular basophilic inclusions and RV in several fibers (Supplementary Figure 1D). Thigh MRI indicated that the semitendinosus, sartorius, and gracilis were the most affected, exceeding the involvement of the anterior compartment. Lower legs MRI displayed the peroneal muscles with significantly more lipomatous changes than those in the tibialis anterior and posterior compartment muscles (Figure 1C). The MRI pattern analysis suggested that Desmin was the causative gene. The gene panel for myopathy detected a heterozygous Desmin mutation c.708C>G, p. (Ile236Met), leading to desminopathy, with a diagnosis supported by lower limb MRI.

##### 3.3.3.2. αB-crystallinopathy

P15 developed weakness in the distal lower legs starting at the age of 26 years. Light microscopy showed the atrophy of muscle fibers with the accumulation of autophagic vacuoles (Supplementary Figure 1E). Muscle MRI revealed focal fatty degeneration of the thigh and calf muscles, with a predominance of the semitendinosus, gracilis, tibialis anterior, and peroneal muscles (Figure 1D). The involvement pattern of the special muscle appeared consistent with the observations in a previous study (11). The gene panel for myopathy yielded a heterozygous *CRYAB* mutation c.31C>T, p. (Arg11Cys), leading to cataract, which is a known phenotype of Arg11Cys-αB-crystallinopathy (12).

##### 3.3.3.3. Filaminopathy

P16 and P17 experienced severe distal lower limb paresis. Histopathological examination revealed subsarcolemmal autophagic vacuoles and abnormal protein aggregates in some muscle fibers (Supplementary Figure 1F). Muscle imaging revealed moderate fatty infiltration in the vastus intermedius, vastus lateralis, semimembranosus, adductor magnus, and long head of the biceps femoris muscles. It also revealed pronounced lipomatous alterations in the soleus, gastrocnemius medialis, and gastrocnemius lateralis muscles (Figure 1E). WES identified a novel 15-nucleotide deletion c.2791_2805del, p. (931_935del) and a missense mutation c.2917G>A, p. (Gly973Ser) in *FLNC*, respectively.

#### 3.3.4. Hereditary myopathy with early respiratory failure (HMERF)

P18 visited the neurology outpatient clinic owing to frequent stumbling on uneven roads and numbness in the lower extremities. Myopathological changes were present as characteristic subsarcolemmal cytoplasmic bodies and RV in the fibers (Supplementary Figures 1G, H). The MRI findings were unique, with fatty degenerative changes in the semitendinosus and anterolateral muscles of the lower legs (Figure 1F). WES analysis detected a mutation c.95358C>G, p. (Asn31786Lys) (previously named as p. Asn30145Lys) in *TTN* (13). Notably, P18 neither had respiratory insufficiency among the presenting symptoms nor developed failure later during the disease course which is frequently encountered in HMERF cases, similar to the findings of a previous report (14). Our clinical findings suggest a comparatively mild phenotype (normal respiratory function) expressed by this variant.

#### 3.3.5. Oculopharyngodistal myopathy (OPDM)

P19.1 had difficulty pronouncing words since the age of 33 years, followed by facial weakness and atrophy of the lower limbs. At the age of 45 years, she had difficulty walking and swallowing. The proband's father (P19.2) had a severe clinical phenotype and presented with ophthalmoplegia and prominent dysphagia in his forties. Her sister (P19.3) complained of bilateral ptosis at the age of 30 years. Her nephew (P19.4) manifested as mild ptosis in his mid-twenties. A biopsy revealed mild subsarcolemmal RV (Supplementary Figure 1I). The soleus and the long head of the biceps femoris were involved, whereas the popliteus, gracilis, and the short head of the biceps femoris were almost spared (Figure 1G). Additionally, autophagic vacuoles, combined with the weakness of the facial and lower limbs, are reminiscent of oculopharyngeal muscular dystrophy (OPMD). However, a normal number of GCG repeats in *PABPN1* was observed, and relevant mutations were not detected using WES. Using a GC-rich polymerase chain reaction, we identified an expansion of 135 CGG repeats at the 5′UTR of *GIPC1* co-segregated in the family.

### 3.4. RVM of proximal muscles weakness with identified pathogenic mutation

#### 3.4.1. Limb-girdle muscular dystrophy D1 (LGMDD1, previously known as LGMD1D)

P20 initially presented with a proximal phenotype, that is, progressive weakness of the proximal muscle, at 40 years of age. Muscle biopsy revealed moderate RV in atrophied muscle fibers (Supplementary Figure 1J). At the age of 56 years, he still continued to walk independently but required a stick for long-distance walking. Leg MRI showed severe fatty infiltration with relative sparing of the gracilis, sartorius, adductor longus, and semitendinosus muscles in the thigh and anterior compartments of calves (Figure 1H). A known mutation of moderate severity, c.298T>C, p. (Phe100Leu), *DNAJB6* variation related to LGMDD1 was discovered (15).

#### 3.4.2. Limb-girdle muscular dystrophy R10 (LGMDR10, previously known as LGMD2I)

P21.1 suffered from mild proximal weakness of the leg muscles starting at the age of 25 years and developed severe foot drop over the age of 20 years. Her brother and two sisters (P21.2, P21.3, and P21.4) had similar complaints at the age of 20 years. The muscle specimen showed central rimmed vacuoles (Supplementary Figure 1K). The vastus internus, gastrocnemius, and soleus muscles revealed advanced dystrophic changes. The vastus lateralis muscle showed severe fatty degeneration on the left side (Figure 1I). The NGS panel for myopathy genes revealed the compound heterozygous mutations c.102328C>T, p. (Arg34110Trp) and c.105201_105202insT, p. Arg34110Trp in *TTN*.

#### 3.4.3. Proximal adult-onset RVM

P22 exhibited a different phenotype: adult-onset limb-girdle weakness and early-onset scoliosis. Histopathological examination revealed the presence of numerous RVs (Supplementary Figure 1L). The semitendinosus, gastrocnemius medialis, and lateralis showed severe fatty degeneration, whereas less severe changes were seen in the adductor longus muscle. The tibialis anterior muscle was relatively spared (Figure 1J). Novel c.61048C>T, p. (Leu20350Phe), and c.58072C>T, p. (Arg19358Cys) *TTN* mutations, which led to missense mutations in the coding sequence, were identified using WES.

#### 3.4.4. Hypokalaemic periodic paralysis (HypoPP)

P23 exhibited plegia of hip flexion and knee extension and a history of aching pain in both legs. Several autophagic vacuoles were observed in a predominantly central position (Supplementary Figure 1M). Muscle involvement patterns showed that the gastrocnemius lateralis, soleus, and gastrocnemius medialis were affected (Figure 1K). These findings of marked autophagy and selectively affected imaging resembled HypoPP caused by dominant mutations in *CACNA1S*. WES did not detect any relevant mutation except for the c.1517G>C, p. (Ser506Thr) variant of unknown significance in exon 11 of *CACNA1S*.

#### 3.4.5. Oculopharyngeal muscular dystrophy (OPMD)

P24.1 and P25 initially presented with ptosis and gradually progressive dysphagia at the age of 50 years. Severe muscle weakness was observed in the proximal muscles at P24.1, whereas P25 had relatively well-preserved arm function. Her mother, P24.2, who showed similar symptoms, lost mobility at the age of 68 years and relied on a wheelchair. Muscle biopsy revealed sporadic RVs (Supplementary Figure 1N). Muscle imaging indicated muscle involvement of the soleus (Figure 1L). We initially suspected OPMD based on slowly progressive bilateral ptosis, proximal-dominant muscle involvement, and family history, suggesting autosomal dominant inheritance. This was confirmed by direct sequencing of *PABPN1*, which revealed insertion variations c.27_28 ins GCGGCGGCAGCA, p.(A11_G12insAAAA), and c.24_c.25 ins GCGGCGGCGGCGGCA.

#### 3.4.6. Congenital myasthenic syndromes (CMS)

P27 developed external ophthalmoplegia 14 years after the diagnosis. Muscle biopsies showed rimmed vacuole alterations, predominantly in the subsarcolemmal position (Supplementary Figure 1O). P27 was categorized as having mild extensive infiltration on both thigh images, and marked fatty changes were detected in both thighs (Figure 1M). WES analysis identified the previously undescribed compound heterozygous mutations c.1428G>C, p. (Lys476Asn) in exon 14 and c.331C>T, p. (Arg111Cys) in exon 4 of *GFPT1*.

### 3.5. Overview of rimmed vacuolar myopathies and patterns of muscle involvement

**Figure 2:**
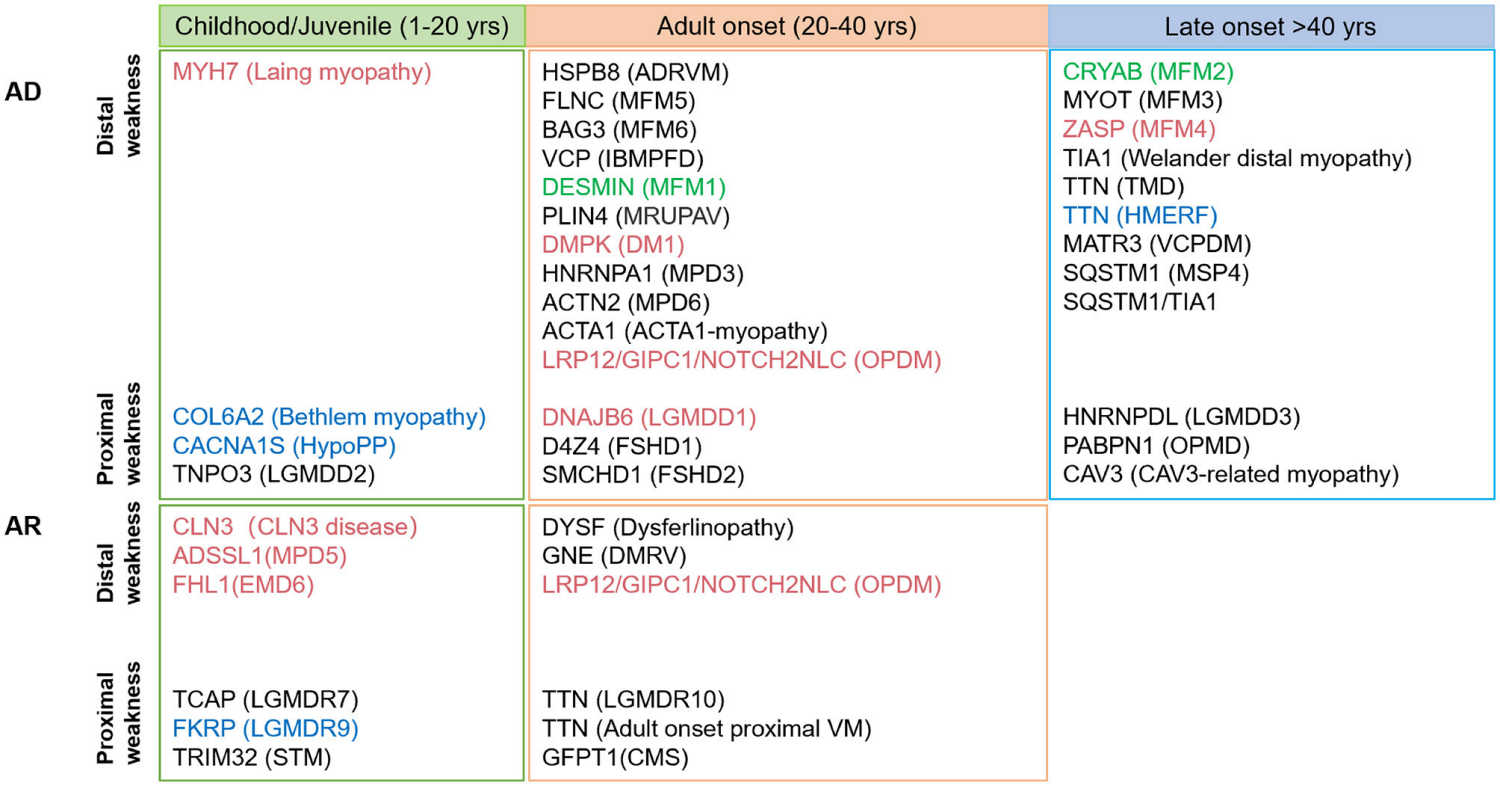
Schematic diagram of the various age (not drawn to scale) at early- and late-onset subtypes of rimmed vacuolar myopathies described based on the hereditary mode. Mutations associated with cardiomyopathy and respiratory failure are shown in red and blue, respectively; mutation corresponding to both is indicated in green.

RV-associated myopathies are summarized in Figure 2 and Supplementary Table 2. The spectrum of RVM is broad, and a schematic of the age distribution of the mutations that cause various diseases is summarized in Figure 2. Currently, ~30 genes of distal weakness (*MYH7, HSPB8, FLNC, BAG3, VCP, DESMIN, DNAJB6, HNRNPA1, ACTN2, ACTA1, NOTCH2NLC, LRP12, GIPC1, CRYAB, MYOT, ZASP, TIA1, TTN, MATR3, SQSTM1, SQSTM1/TIA1*, and *PLIN4*) and proximal weakness *(COL6A2, CACNA1S, TNPO3, D4Z4, SMCHD1, HNRNPDL, PABPN1*, and *CAV3*) are associated with the autosomal dominant (AD) form of RVM. Pathogenic changes in 13 genes associated with distal weakness (*CLN3, ADSSL1, FHL1, DYSF, GNE, NOTCH2NLC, LRP12*, and *GIPC1*) and proximal weakness (*TCAP, FKRP, TRIM32, TTN*, and *GFPT1*) cause an autosomal recessive (AR) form of RVM, and disease-causing variants in four genes (*NOTCH2NLC, LRP12, GIPC1*, and *TTN*) result in either AD or AR RVM.

**Figure 3:**
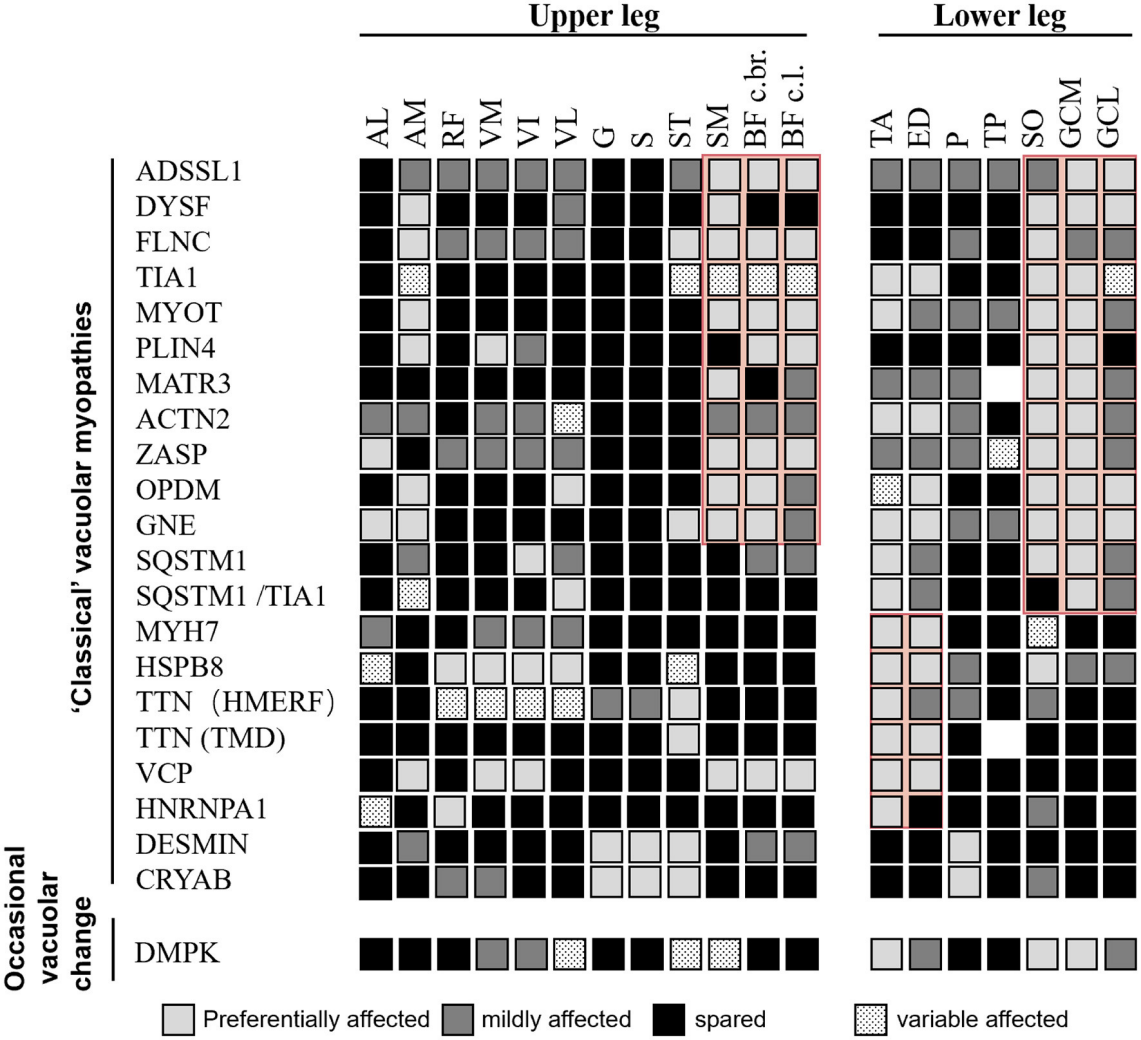
Literature-based pattern of rimmed vacuolar myopathies with distal weakness. Both “classical” vacuolar myopathies (consistent vacuolar phenotype) and myopathies with occasional vacuolar changes are included. Disease entities showing relevant similarities are highlighted in red. Modified from Mensch et al. (24).

**Figure 4:**
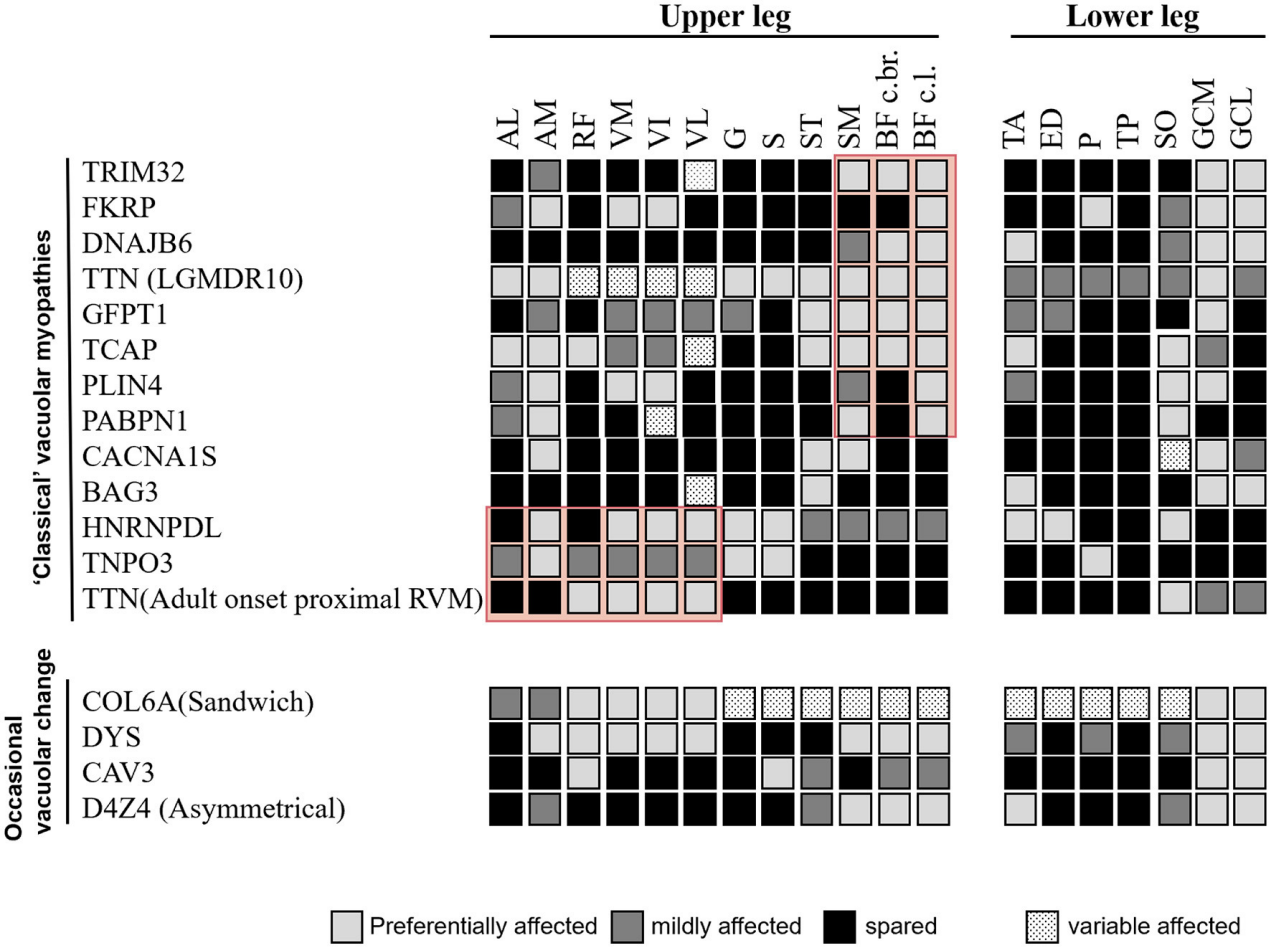
Comparison of the literature-based pattern between rimmed vacuolar myopathies with proximal weakness in different disease entities.

Comprehensive information regarding disease-specific patterns is lacking for RVM because only a few reports have relied on a small number of individuals studied. Based on the available literature, we reviewed the literature-based lower limb MRI patterns of RVM (Figures 3, 4) and found a pattern of predominant fatty replacement of the posterior and anterior compartments.

## 4. Discussion

RVMs are difficult to differentiate clinically because of the shared characteristics of weakness patterns. Muscle imaging is an indispensable tool for the diagnosing workup of patients with muscular dystrophies and distal myopathies (16, 17). However, the number of reports on MRI of RVM, including systematic literature reviews, is limited. Therefore, a comprehensive MRI pattern analysis of RVM was performed. To the best of our knowledge, this is the first study to summarize the imaging features of RVMs.

A predominant effect on the distal or proximal lower limbs was observed, corresponding to the patient's initial symptoms. The diseases were classified into four groups based on their distinct imaging patterns (Figures 2–4). The typical imaging characteristics of predominant involution of the posterior lower leg muscle were captured in the first group, which started with a distal affection. Our analysis identified imaging differences between groups of patients with childhood-onset RVM variants of *ADSSL1* (18); relatively early-onset RVM caused by *FLNC* and *DYSF* mutations (19, 20); and late-onset subgroups associated with mutations in *TIA1, MYOT, PLIN4, MATR3, ACTN2, SQSTM1, SQSTM1/TIA1, GNE, GIPC1, LRP12*, and *NOTCH2NLC* (21–29). In terms of fatty infiltration, all patients showed a combination of dominant involvement of the muscles in the posterior compartment of the leg and posterior compartment of the thigh. Overall, *ADSSL1* myopathy, Welander myopathy, myotilinopathy, *MATR3* mutations associated with vocal cord and pharyngeal distal myopathy, filaminopathy, and *MYOT*-related myopathy appear to have a selective effect on parts of the hamstring muscles (semimembranosus and the long head of the biceps femoris muscles), while the anterior compartments are largely spared (24). Dysferlinopathy, another disease characterized by calf muscle involvement, results in preferred involvement of the legs (gastrocnemius medialis and soleus) and thighs (semimembranosus, semitendinosus, and adductor magnus) (20). Patients with *ACTN2* mutations tend to have a complete fatty replacement of the anterolateral compartment muscles of the lower legs, but mostly sparing the thigh muscles (26). Muscle scans of patients with *SQSTM1/TIA1* mutations revealed fatty replacement in the adductor magnus and vastus lateralis of the thighs and the tibialis anterior and medial gastrocnemius of the lower limbs (21). Patients with GNE myopathy have a similar pattern of muscle involvement as patients with OPDM, with predominant involvement of soleus and gastrocnemius medialis even in the early stages (27, 30). However, some differences were observed: in GNE myopathy, the tibialis anterior dysfunction and vastus lateralis are spared, whereas, in OPDM, the long head of the biceps femoris, popliteus, and the short head of the biceps femoris are relatively spared (28).

The second group showed that the dominant anterior compartment muscles were involved, including childhood-onset RVM caused by *MYH7* mutations; adult early-onset RVM variants in *HSPB8, HNRNPA*, and *Desmin*; and late-onset subtypes related to mutations in *CRYAB, TTN*, and *VCP*. Furthermore, the tibialis anterior muscle can be considered as a “red flag” indicating an *MYH7*-related myopathy (31).

The third group was characterized by muscle weakness that was more pronounced proximal than distal. When a posterior-dominant lower limb muscle involvement pattern is observed, the possibility of childhood-onset RVM caused by *TRIM32* and *FKRP* mutations, relatively early-onset RVM associated with mutations in *DNAJB6, TTN, GFPT1, TCAP*, and *D4Z4*, and late-onset subtype variants in *PABPN1* should be noted. Concentric fatty infiltration (in the vastus intermedius and vastus medialis muscles, with relative sparing of the rectus femoris, vastus lateralis, and biceps femoris short head muscles) has been observed in LGMDR9 (17). Although the muscle involvement patterns of these diseases are similar, the gastrocnemius muscle, which is commonly spared in OPMD, can be a clue for the differential diagnosis (32).

Patients in the fourth group had more pronounced proximal anterior thigh muscle weakness, including relatively early-onset RVM caused by *HNRNPDL, TNPO3*, and *TTN* mutations (33, 34). In addition, marked autophagy impairment has been linked to known pathogenic variants of genes not regularly associated with autophagic defects in myotonic dystrophy type 1, Becker muscular dystrophy, spinal and bulbar muscular atrophy, and collagen VI-related Bethlem myopathy (35–37), which further expands the spectrum of RVM.

Moreover, our findings demonstrated that MRI analysis could support this diagnostic process. The review of the currently available literature revealed that MRI pattern analysis suggested a limited number of causative genes for screening pathogenic mutations by focusing on the recognition of key features reported to be characteristic of each gene. Using our template, we correctly identified all three patients (P14, P15, and P18), who presented with preferential involvement of the semitendinosus and peroneal muscles suggestive of Desmin-associated myopathy, *CRYAB*-associated myopathy, and HMERF, respectively. Additionally, eight of 12 patients with GNE myopathy were correctly identified. In four patients, the MRI pattern did not suggest the correct diagnosis. However, they showed a prominent involvement of the short head of the biceps femoris muscle, suggesting that this could also serve as a key feature, as reported previously (38). Therefore, the emergence of MRI may shorten the diagnostic process and provide valuable clues for differential diagnosis, regardless of the similarity or variability of clinical symptoms.

In the current NGS era, the usage of MRI in single genetic testing is limited. However, the use of MRI pattern analysis in suggesting a limited number of candidate genes per participant may be beneficial in supporting NGS analysis when unknown significance is detected. In our cohort, for example, P22 carrying the novel c.61048C>T (p. Leu20350Phe) and c.58072C>T (p. Arg19358Cys) *TTN* mutations has an MRI pattern of severe fatty degeneration in the semitendinosus, gastrocnemius medialis, and lateralis and less severe changes in the adductor longus muscle suggestive of adult-onset proximal rimmed vacuolar titinopathy (39, 40). As demonstrated in this case, MRI pattern analysis supports the pathogenicity of novel mutations detected by NGS and eventually resolves an uncertain genetic diagnosis.

Collectively, our findings refined the clinical features, muscle imaging, and pathological data of the four subtypes of RVM and revealed the distinction between distal and proximal weakness. Thus, we confirmed the differential topography of previously reported muscle involvement (11, 41). However, this study had some limitations. Muscle scanning can be less productive in asymptomatic instances or cases in the earliest clinical stages of the disease because it might not detect any alteration or mild change in a single muscle, which is insufficient for correct distinction. Furthermore, patients in very advanced stages with wide fatty replacement (i.e., both distal and proximal muscles affected) may not show distinctive imaging findings. In conclusion, caution must be applied, and genes must not be eliminated solely based on MRI pattern analysis.

## Data availability statement

The raw data supporting the conclusions of this article will be made available by the authors, without undue reservation.

## Ethics statement

The studies involving human participants were reviewed and approved by the Ethics Committee from the First Hospital of Jilin University, Changchun, China. Written informed consent to participate in this study was provided by the participants' legal guardian/next of kin.

## Author contributions

X-jW, HS, JM, R-qQ, Z-zJ, Z-wM, WS, and X-fY contributed to the study design and critical revision of the manuscript. X-jW, HS, JM, R-qQ, and X-fY contributed to data acquisition and analysis. X-jW, HS, JM, and R-qQ contributed to manuscript drafting. All authors edited and approved the final version of the manuscript.

## Supplementary material

Supplementary Figure 1 Rimmed vacuoles in rimmed vacuolar myopathies. **(A, B)** Numerous subsarcolemmal rimmed vacuoles (white arrow) in GNE myopathy (P1). **(C)** Subsarcolemmal rimmed vacuoles (white arrow) in P12 with dysferlinopathy. **(C)** Atrophy muscle fibers with accumulations of autophagic vacuoles (white arrow) in the biopsy of P14. **(D)** Branching granular basophilic inclusions (black arrow) and RV (white arrow). **(E)** Moderate RV (white arrow) in atrophy muscle fibers. **(F)** Prominent autophagic vacuoles (white arrow) combined with polymorphous inclusions (black arrow). **(G, H)** Subsarcolemmal cytoplasmic bodies (black arrow) and RV in fibers of P18. **(I)** Subsarcolemmal RV (white arrow). **(J)** Muscle biopsy found moderate RV (white arrow) in atrophy muscle fibers. **(K)** Central rimmed vacuoles (white arrow). **(L)** Histopathological revealed mild-rimmed vacuoles (white arrow). **(M)** Several autophagic vacuoles (white arrow) in a predominantly subsarcolemmal position. **(N)** Muscle biopsy revealed sporadic rimmed vacuoles (white arrow).

Supplementary Table 1 Clinical characteristics of 36 patients with rimmed vacuolar myopathies.

Supplementary Table 2 Summary of hereditary rimmed vacuolar myopathies in the literature.

## References

[1] Mair D Biskup S Kress W Abicht A Brück W Zechels S. Differential diagnosis of vacuolar myopathies in the NGS era. Brain Pathol. (2020) 30:877–96. 10.1111/bpa.12864 32419263 PMC8017999

[2] Wattjes MP Kley RA Fischer D. Neuromuscular imaging in inherited muscle diseases. Eur Radiol. (2010) 20:2447–60. 10.1007/s00330-010-1799-2 20422195 PMC2940021

[3] Morrow JM Sinclair CD Fischmann A Machado PM Reilly MM Yousry TA. MRI biomarker assessment of neuromuscular disease progression: a prospective observational cohort study. Lancet Neurol. (2016) 15:65–77. 10.1016/S1474-4422(15)00242-2 26549782 PMC4672173

[4] Zheng Y Li W Du J Jin S Li S Zhao Y. The trefoil with single fruit sign in muscle magnetic resonance imaging is highly specific for dystrophinopathies. Eur J Radiol. (2015) 84:1992–8. 10.1016/j.ejrad.2015.06.011 26119801

[5] Xie Z Xiao J Zheng Y Wang Z. Yuan Y. Magnetic resonance imaging findings in the muscle tissue of patients with limb girdle muscular dystrophy type 2I harboring the founder mutation c545A>G in the FKRP. Gene BioMed Res Int. (2018) 2018:3710814. 10.1155/2018/3710814 30003095 PMC5996470

[6] Miao J Su FF Liu XM Wei XJ Yuan Y Yu XF. case report: a heterozygous deletion (2791_2805 del) in exon 18 of the filamin C gene causing filamin C-related myofibrillar myopathies in a Chinese family. BMC Neurol. (2018) 18:79. 10.1186/s12883-018-1078-4 29866061 PMC5985593

[7] Wei XJ Miao J Kang ZX Gao YL Wang ZY Yu XF A. novel homozygous exon2 deletion of TRIM32 gene in a Chinese patient with sarcotubular myopathy: a case report and literature review. Bosn J Basic Med Sci. (2021) 21:495–500. 10.17305/bjbms.2020.5288 33485293 PMC8292861

[8] Ulintz PJ Wu W Gates CM. Bioinformatics analysis of whole exome sequencing data. Methods Mol Biol. (2019) 1881:277–318. 10.1007/978-1-4939-8876-1_21 30350213

[9] Su F Miao J Liu X Wei X Yu X. Distal myopathy with rimmed vacuoles: spectrum of GNE gene mutations in seven Chinese patients. Exp Ther Med. (2018) 16:1505–12. 10.3892/etm.2018.6344 30112071 PMC6090448

[10] Miao J Wei XJ Wang X Yin X Yu XF. A case report: identification of a novel exon 1 deletion mutation in the GNE gene in a Chinese patient with GNE myopathy. Medicine. (2020) 99:e22663. 10.1097/MD.0000000000022663 33031330 PMC7544422

[11] Fischer D Kley RA Strach K Meyer C Sommer T Eger K. Distinct muscle imaging patterns in myofibrillar myopathies. Neurology. (2008) 71:758–65. 10.1212/01.wnl.0000324927.28817.9b 18765652 PMC2583436

[12] Jiao X Khan SY Irum B Khan AO Wang Q Kabir F. Missense mutations in CRYAB are liable for recessive congenital cataracts. PLoS ONE. (2015) 10:e0137973. 10.1371/journal.pone.0137973 26402864 PMC4581838

[13] Pfeffer G Barresi R Wilson IJ Hardy SA Griffin H Hudson J. Titin founder mutation is a common cause of myofibrillar myopathy with early respiratory failure. J Neurol Neurosurg Psychiatry. (2014) 85:331–8. 10.1136/jnnp-2012-304728 23486992 PMC6558248

[14] Palmio J Leonard-Louis S Sacconi S Savarese M Penttila S Semmler AL. Expanding the importance of HMERF titinopathy: new mutations and clinical aspects. J Neurol. (2019) 266:680–90. 10.1007/s00415-019-09187-2 30666435 PMC6394805

[15] Ruggieri A Brancati F Zanotti S Maggi L Pasanisi M Saredi S. Complete loss of the DNAJB6 G/F domain and novel missense mutations cause distal-onset DNAJB6 myopathy. Acta Neuropathol Commun. (2015) 3:44. 10.1186/s40478-015-0224-0 26205529 PMC4513909

[16] Bugiardini E Morrow JM Shah S Wood CL Lynch DS Pitmann AM. The diagnostic value of MRI pattern recognition in distal myopathies. Front Neurol. (2018) 9:456. 10.3389/fneur.2018.00456 29997562 PMC6028608

[17] Xie Z Xie Z Yu M Zheng Y Sun C Liu Y. Value of muscle magnetic resonance imaging in the differential diagnosis of muscular dystrophies related to the dystrophin-glycoprotein complex. Orphanet J Rare Dis. (2019) 14:250. 10.1186/s13023-019-1242-y 31747956 PMC6865054

[18] Park HJ Hong YB Choi YC Lee J Kim EJ Lee JS. ADSSL1 mutation relevant to autosomal recessive adolescent onset distal myopathy. Ann Neurol. (2016) 79:231–43. 10.1002/ana.24550 26506222

[19] Kley RA Serdaroglu-Oflazer P Leber Y Odgerel Z van der Ven PF Olivé M. Pathophysiology of protein aggregation and extended phenotyping in filaminopathy. Brain. (2012) 135:2642–60. 10.1093/brain/aws200 22961544 PMC3437028

[20] Diaz-Manera J Fernandez-Torron R J LL James MK Mayhew A Smith FE. Muscle MRI in patients with dysferlinopathy: pattern recognition and implications for clinical trials. J Neurol Neurosurgery Psychiatry. (2018) 89:1071–81. 10.1136/jnnp-2017-317488 29735511 PMC6166612

[21] Niu Z Pontifex CS Berini S Hamilton LE Naddaf E Wieben E. Myopathy with SQSTM1 and TIA1 variants: clinical and pathological features. Front Neurol. (2018) 9:147. 10.3389/fneur.2018.00147 29599744 PMC5868303

[22] Olivé M Odgerel Z Martínez A Poza JJ Bragado FG Zabalza RJ. Clinical and myopathological evaluation of early- and late-onset subtypes of myofibrillar myopathy. Neuromuscul Disord. (2011) 21:533–42. 10.1016/j.nmd.2011.05.002 21676617 PMC5148150

[23] Yang K Zeng YH Qiu YS Lin F Chen HZ Jin M. Expanding the phenotype and genotype spectra of PLIN4-associated myopathy with rimmed ubiquitin-positive autophagic vacuolation. Acta Neuropathol. (2022) 143:733–5. 10.1007/s00401-022-02422-7 35499779

[24] Mensch A Kraya T Koester F Muller T Stoevesandt D Zierz S. Whole-body muscle MRI of patients with MATR3-associated distal myopathy reveals a distinct pattern of muscular involvement and highlights the value of whole-body examination. J Neurol. (2020) 267:2408–20. 10.1007/s00415-020-09862-9 32361838 PMC7358922

[25] Bucelli RC Arhzaouy K Pestronk A Pittman SK Rojas L Sue CM. SQSTM1 splice site mutation in distal myopathy with rimmed vacuoles. Neurology. (2015) 85:665–74. 10.1212/WNL.0000000000001864 26208961 PMC4553032

[26] Savarese M Vihola A Jokela ME Huovinen SP Gerevini S Torella A. Out-of-frame mutations in ACTN2 last exon cause a dominant distal myopathy with facial weakness. Neurol Genet. (2021) 7:e619. 10.1212/NXG.0000000000000619 34386585 PMC8356702

[27] Liu CY Yao J Kovacs WC Shrader JA Joe G Ouwerkerk R. Skeletal muscle magnetic resonance biomarkers in GNE myopathy. Neurology. (2021) 96:e798–808. 10.1212/WNL.0000000000011231 33219145 PMC7884988

[28] Zhao J Liu J Xiao J Du J Que C Shi X. Clinical and muscle imaging findings in 14 mainland chinese patients with oculopharyngodistal myopathy. PLoS ONE. (2015) 10:e0128629. 10.1371/journal.pone.0128629 26039504 PMC4454561

[29] Kumutpongpanich T Ogasawara M Ozaki A Ishiura H Tsuji S Minami N. Clinicopathologic features of oculopharyngodistal myopathy with LRP12 CGG repeat expansions compared with other oculopharyngodistal myopathy subtypes. JAMA Neurol. (2021). 10.1001/jamaneurol.2021.1509 34047774 PMC8164150

[30] Fatehi F Advani S Okhovat AA Ziaadini B Shamshiri H Nafissi S. Thigh and leg muscle MRI findings in GNE myopathy. J Neuromuscl Dis. (2021) 8:735–42. 10.3233/JND-210629 34334416

[31] Fiorillo C Astrea G Savarese M Cassandrini D Brisca G Trucco F. MYH7-related myopathies: clinical, histopathological and imaging findings in a cohort of Italian patients. Orphanet J Rare Dis. (2016) 11:91. 10.1186/s13023-016-0476-1 27387980 PMC4936326

[32] Mirabella M Silvestri G de Rosa G Di Giovanni S Di Muzio A Uncini A. GCG genetic expansions in Italian patients with oculopharyngeal muscular dystrophy. Neurology. (2000) 54:608–14. 10.1212/WNL.54.3.608 10680791

[33] Berardo A Lornage X Johari M Evangelista T Cejas C Barroso F. HNRNPDL-related muscular dystrophy: expanding the clinical, morphological and MRI phenotypes. J Neurol. (2019) 266:2524–34. 10.1007/s00415-019-09437-3 31267206

[34] Pál E Zima J Hadzsiev K Ito YA Hartley T Boycott KM. A novel pathogenic variant in TNPO3 in a Hungarian family with limb-girdle muscular dystrophy 1F. Eur J Med Genet. (2019) 62:103662. 10.1016/j.ejmg.2019.05.001 31071488

[35] Ban R Zhang Y Li K Shi Q A. Case of myotonic dystrophy type i with rimmed vacuoles in skeletal muscle pathology. J Clin Rheumatol. (2020). 10.1097/RHU.0000000000001496 32732524

[36] Momma K Noguchi S Malicdan MC Hayashi YK Minami N Kamakura K. Rimmed vacuoles in Becker muscular dystrophy have similar features with inclusion myopathies. PLoS One. (2012) 7:e52002. 10.1371/journal.pone.0052002 23251671 PMC3522649

[37] Klickovic U Zampedri L Sinclair CDJ Wastling SJ Trimmel K Howard RS. Skeletal muscle MRI differentiates SBMA and ALS and correlates with disease severity. Neurology. (2019) 93:e895–907. 10.1212/WNL.0000000000008009 31391248 PMC6745729

[38] Tasca G Ricci E Monforte M Laschena F Ottaviani P Rodolico C. Muscle imaging findings in GNE myopathy. J Neurol. (2012) 259:1358–65. 10.1007/s00415-011-6357-6 22231866

[39] Evilä A Arumilli M Udd B Hackman P. Targeted next-generation sequencing assay for detection of mutations in primary myopathies. Neuromuscul Disord. (2016) 26:7–15. 10.1016/j.nmd.2015.10.003 26627873

[40] Evilä A Vihola A Sarparanta J Raheem O Palmio J Sandell S. Atypical phenotypes in titinopathies explained by second titin mutations. Ann Neurol. (2014) 75:230–40. 10.1002/ana.24102 24395473

[41] Schramm N Born C Weckbach S Reilich P Walter MC Reiser MF. Involvement patterns in myotilinopathy and desminopathy detected by a novel neuromuscular whole-body MRI protocol. Eur Radiol. (2008) 18:2922–36. 10.1007/s00330-008-1071-1 18648820

