# Supplementary figures and images for: Clinical-pathological features and muscle imaging findings in 36 Chinese patients with rimmed vacuolar myopathies: case series study and review of literature
Source: Front Neurol. 2023 Apr 28;14:1152738. doi: 10.3389/fneur.2023.1152738 (PMC10175607; doi:10.3389/fneur.2023.1152738)

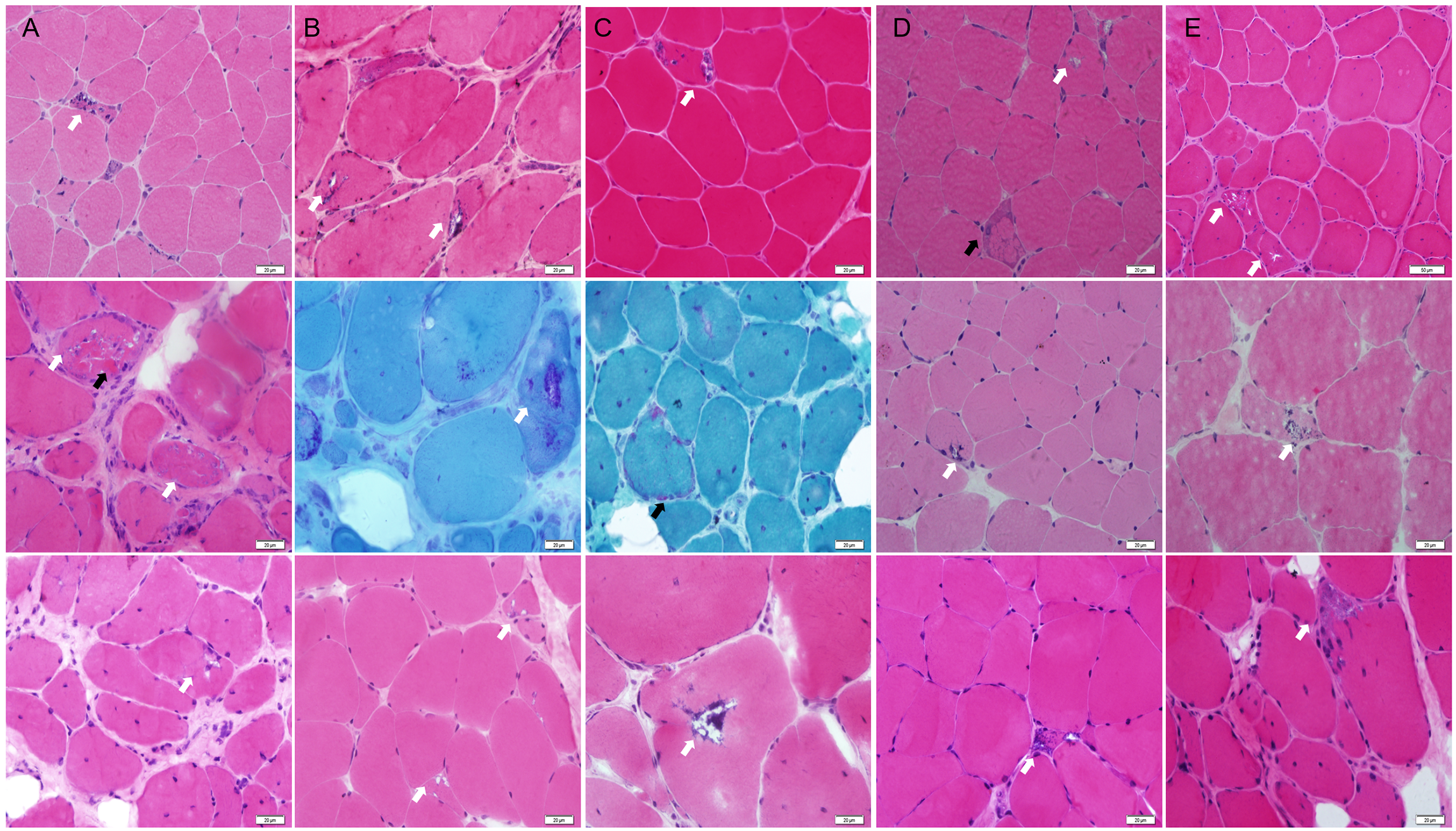

Supplement: Supplementary Figure 1 — Rimmed vacuoles in rimmed vacuolar myopathies. (A, B) Numerous subsarcolemmal rimmed vacuoles (white arrow) in GNE myopathy (P1). (C) Subsarcolemmal rimmed vacuoles (white arrow) in P12 with dysferlinopathy. (C) Atrophy muscle fibers with accumulations of autophagic vacuoles (white arrow) in the biopsy of P14. (D) Branching granular basophilic inclusions (black arrow) and RV (white arrow). (E) Moderate RV (white arrow) in atrophy muscle fibers. (F) Prominent autophagic vacuoles (white arrow) combined with polymorphous inclusions (black arrow). (G, H) Subsarcolemmal cytoplasmic bodies (black arrow) and RV in fibers of P18. (I) Subsarcolemmal RV (white arrow). (J) Muscle biopsy found moderate RV (white arrow) in atrophy muscle fibers. (K) Central rimmed vacuoles (white arrow). (L) Histopathological revealed mild-rimmed vacuoles (white arrow). (M) Several autophagic vacuoles (white arrow) in a predominantly subsarcolemmal position. (N) Muscle biopsy revealed sporadic rimmed vacuoles (white arrow). [file Image_1.tif]
