# Supplementary material for: Clinical-pathological features and muscle imaging findings in 36 Chinese patients with rimmed vacuolar myopathies: case series study and review of literature
Source: Front Neurol. 2023 Apr 28;14:1152738. doi: 10.3389/fneur.2023.1152738 (PMC10175607; doi:10.3389/fneur.2023.1152738)
Supplement: Supplementary Table 1 — Clinical characteristics of 36 patients with rimmed vacuolar myopathies. [file Data_Sheet_1.pdf]

Supplementary Table 1 Clinical characteristics of 36 patients with rimmed vacuolar myopathies.

Patient No.	sex	Age	Gene	Mutation, DNA		Mutation, Protein		Muscle phenotype	CK(U/L)
1.1	F	45	GNE	c.1426A>G	c.1807G>C	p. Arg476Gly	p. Val603Leu	Distal > proximal weakness	602
1.2	M	42	GNE	c.1426A>G	c.1807G>C	p. Arg476Gly	p. Val603Leu	Distal > proximal weakness	876
2	F	44	GNE	c.1262C>T	Hom	p. Pro421Leu		Distal > proximal weakness	699
3	M	46	GNE	c.620A>T	Hom	p. Asp207Val		Distal > proximal weakness	Normal
4.1	M	45	GNE	c.1262C>T	c.859G>A	p. Pro421Leu	p. Ala287Thr	Distal > proximal weakness	777
4.2	F	43	GNE	c.1262C>T	c.859G>A	p. Pro421Leu	p. Ala287Thr	Distal > proximal weakness	698
5	F	32	GNE	c.620A>T	Exon1 del	p. Asp207Val		Distal > proximal weakness	458
6	M	26	GNE	c.131G>C	c.1726G>C	p. Cys44Ser	p. Gly576Arg	Distal > proximal weakness	578
7	M	43	GNE	c.620A>T	c.2005G>C	p. Asp207Val	p. Ala669Pro	Distal > proximal weakness	487
8	F	34	GNE	c.620A>T	c.653A>G	p. Asp207Val	p. Asp218Gly	Distal > proximal weakness	568
9	F	35	GNE	c.455_456insC	het			Distal > proximal weakness	632
10	F	46	GNE	c.131G>C	het	p. Cys44Ser		Distal > proximal weakness	254
11	M	27	DYSF	c.dup1471 A	Hom	p. Met491AsnfsTer15		Distal > proximal weakness	3783
12	M	30	DYSF	c.3866delG	c.4106delT	p. Trp1289CysfsTer4	p. Leu1369ArgfsTer8	Distal > proximal weakness	8823
13	F	33	DYSF	c.4321C>T	c.2875 C>T	p. Gln1441X	p. Arg 959Trp	Distal > proximal weakness	1756
14	F	43	DESMIN	c.708C>G	het	p. Ile236Met		Distal > proximal weakness	314
15	M	31	CRYAB	c.31C>T	het	p. Arg11Cys		Distal > proximal weakness	1752
16	F	42	FLNC	c.2791_2805del	het	p.931_935del		Distal > proximal weakness	467
17	M	51	FLNC	c.2917G>A	het	p. Gly973Ser		Distal > proximal weakness	734
18	M	43	TTN	c.95358C>G	het	p. Asn31786Lys		Distal > proximal weakness	375
19.1	F	41	GIPC1	CGG 135				Distal > proximal weakness	546

19.2	M	63	GIPC1	CGG 135				Distal > proximal weakness	n.d.
19.3	F	38	GIPC1	CGG 135				Distal > proximal weakness	n.d.
19.4	M	16	GIPC1	CGG 135				Distal > proximal weakness	n.d.
P20	M	53	DNAJB6	c.298T>C	het	p. Phe100Leu		Proximal > distal weakness	782
P21.1	M	53	TTN	c.102328C>T	c.105201_105202insT	p. Arg34110Trp	p. Lys35068Ter	Proximal > distal weakness	Normal
P21.2	F	52	TTN	c.102328C>T	c.105201_105202insT	p. Arg34110Trp	p. Lys35068Ter	Proximal > distal weakness	n.d.
P21.3	F	50	TTN	c.102328C>T	c.105201_105202insT	p. Arg34110Trp	p. Lys35068Ter	Proximal > distal weakness	n.d.
P21.4	F	47	TTN	c.102328C>T	c.105201_105202insT	p. Arg34110Trp	p. Lys35068Ter	Proximal > distal weakness	n.d.
P22	F	19	TTN	c.61048C>T	c.58072C>T	p. Leu20350Phe	p. Arg19358Cys	Proximal > distal weakness	503
P23	M	53	CACNA1S	c.1517G>C	het	p. (Ser506Thr)		Proximal > distal weakness	734
P24.1	F	63	PABPN1	c.27-28 ins GCGGCGGCAGCA		p.A11_G12insAAAA		Proximal > distal weakness	323
P24.2	F	83	PABPN1	c.27-28 ins GCGGCGGCAGCA		p.A11_G12insAAAA		Proximal > distal weakness	n.d.
P25	F	54	PABPN1	c.24_c.25 ins GCGGCGGCGGCGGCA		p. Ala7_Ala11 dup		Proximal > distal weakness	202
P26	M	50	TRIM32	Exon2 del	hom			Proximal > distal weakness	403
P27	M	23	GFPT1	c.1428G>C	c.331C>T	p. Lys476Asn	p. Arg111Cys	Proximal > distal weakness	836

F= female; M= male; Hom. = homozygous; Het. = heterozygous; n.r.= not reported; n.d. = not done; CK = creatine kinase.

Supplementary Table 2 Summary of hereditary rimmed vacuolar myopathies in the literature.

Disease entities	Gene defects	Causes Cases of this study References	Cardiac involvement	Respiratory involvement	Muscle MRI	Muscle Pathology
**Rimmed vacuolar myopathies with distal weakness**						
Myofibrillar myopathy	*DESMIN(MFM1)*	P14, (1, 2)	+	+	G, S, ST (thigh); P(calf)	RV, "Rubbed-out" fibers
	CRYAB(MFM2)	P15, (2, 3)	+	+	G, S, ST (thigh); P(calf)	RV, granulofilamentous material accumulation
	MYOT(MFM3)	(2)	-	-	AM, ST, SM, BF (thigh); TA, SO, GCM (calf)	RV, hyaline inclusions
	ZASP(MFM4)	(2, 4)	+	-	AL, SM, BF (thigh); TP, SO, GCM (calf)	RV, hyaline inclusions
	FLNC(MFM5)	P16, P17, (5)	-	-	AM, ST, SM, BF (thigh); SO (calf)	RV, polymorphous inclusions
	BAG3(MFM6)	(6, 7)	-	-	VL, ST (thigh); TA, GCM, GCL (calf)	RV, cytoplasmic bodies
Distal myopathy	*GNE* (GNE myopathy)	P1-P10 (8)	-	-	AL, AM, ST, SM, BF c.br. (thigh); TA, ED, SO, GCM, GCL (calf)	RV
	TIA1 (Welander)	(9)	-	-	AM, ST, SM, BF (thigh); TA, ED, SO, GCM, GCL (calf)	RV
	SQSTM1	(10)	-	-	BF (thigh); TA, SO, GCM (calf)	RV
	SQSTM1/TIA1	(11)	-	-	AM, VL, BF (thigh); TA, SO, (calf)	RV
	TTN (Udd)	(12)	-	-	ST (thigh); TA, ED (calf)	RV
	TTN (HMERF)	P18, (13)	-	+	ST (thigh); TA, ED (calf)	RV, cytoplasmic bodies

	DYSF (Dysferlinopathy)	P11-P13, (14)	-	-	AM, SM (thigh); SO, GCM, GCL (calf)	RV, dysferlin defect
	MYH7 (Laing)	(15)	-	-	AL, VM, VI, VL (thigh); TA, ED, SO (calf)	RV, cores
	GIPC1, LRP12, NOTCH2NLC (OPDM)	P19, (16, 17)	-	-	AM, SM, BFc.br. (thigh); ED, SO, GCM, GCL (calf)	RV,Intranuclear tubulofilamentous inclusions
vacuolar myopathy and motor neurone disease	*HSPB8* (ADRVM)	(18, 19)	-	-	RF, VM, VI, VL, ST (thigh); TA, ED, SO (calf)	RV
	VCP (IBMPFD, ALS)	(20, 21)	-	-	AM, VM, VI, SM, BF (thigh); TA, ED (calf)	RV
	hnRNPA2B1, hnRNPA1	(22)	-	-	AL, RF (thigh); TA (calf)	RV
	MATR3 (VCPDM, ALS)	(23)	-	-	SM (thigh); SO, GCM (calf)	RV
**Rimmed vacuolar myopathies with distal weakness with occasional vacuolar changes**						
Myotonic dystrophy type 1	*DMPK*	(24)	+	+	VL, ST, SM (thigh); TA, SO, GCM (calf)	RV, sarcoplasmic masses
vacuolar myopathy and motor neurone disease	*DNM2(CMT)*	(25)	-	-	Intact(thigh); TA (calf)	RV, predominance of type 2 fibers.
**Rimmed vacuolar myopathies with proximal weakness**						
Neuronal ceroid lipofuscinosis	*CLN3(NCL3 disease)*	(26)	+	-	n.r.	RV, curvilinear bodies
Muscular dystrophy	*DNAJB6* (LGMDD1)	P20, (27, 28)	+	-	BF (thigh); TA, GCM, GCL (calf)	RV, cytoplasmic inclusions
	TNPO3(LGMDD2)	(29)	-	-	AM, G, S (thigh); P (calf)	RV, cytoplasmic bodies
	hnRNPDL (LGMDD3)	(30, 31)	-	-	AM, VM, VI, VL, G, S (thigh); TA, ED, SO (calf)	RV
	TCAP(LGMDR7)	(32, 33)	-	-	AL, AM, RF, VL, ST, SM, BF (thigh); TA, SO (calf)	RV

	FKRP(LGMDR9)	(34)	-	+	AM, VM, VI, BFc.l. (thigh); P, GCM, GCL (calf)	RV
	TTN(LGMDR10)	P21, (12)	-	-	All	RV
	TRIM32 (STM)	P26, (35)	-	-	VL, SM, BF (thigh); GCM, GCL (calf)	RV, sarcotubular alterations
	PLIN4(MRUPAV)	(36)	-	-	AM, VM, VI, BFc.l. (thigh); P, SO, GCM (calf)	RV, membranous bodies
	PABPN1 (OPMD)	P24, P25, (37)	-	+	AM, VI, SM, BFc.l. (thigh); SO (calf)	RV, intranuclear thin tubulofilaments
Hypokalaemic periodic paralysis	*CACNA1S, SCN4A*	P23, (38, 39)	-	+	AM, ST, SM (thigh); SO, GCM (calf)	sarcoplasmic reticulum vacuoles
Proximal adult-onset rimmed vacuolar myopathy	*TTN*	P22, (12, 40)	-	-	RF, VM, VI, VL (thigh); SO (calf)	RV
Reducing body myopathy	*FHL1*	(41)	-	-	AM, ST, SM, BF (thigh);	RV, reducing bodies
congenital myasthenic syndromes	*GFPT1(CMS)*	P27, (42)	-	-	ST, SM, BF (thigh); GCM (calf)	RV, tubular aggregates
**Rimmed vacuolar myopathies with proximal weakness with occasional vacuolar changes**						
Bethlem myopathy	*COL6A2*	(43)	-	+	RF(Target),**"Sandwich"**	RV, mitochondrial alterations
Muscular dystrophy	*D4Z4* (FSHD)	(44)	-	-	SM, BF (thigh); "**Asymmetrical"** TA, GCM, GCL (calf)	RV, inflammatory infiltration
Myosin related myopathy	*MYH2*	(45)	-	-	VL, G, ST (thigh);	RV, hyaline bodies
Nemaline-body myopathy	*ACTA1*	(46)	-	-	ST (thigh); TA, ED (calf)	RV
CAV-3 related myopathy	*CAV3*	(47)	-	-	RF, S (thigh); GCM, GCL (calf)	RV, membrane loops
Becker Muscular Dystrophy	*DYS(BMD)*	(48)	+	-	"The trefoil with single fruit sign"	RV
vacuolar myopathy and motor neurone disease	*AR(*SBMA*)*	(49)	-	-	diffuse (thigh)	RV, neurogenic

Legend Tab. 2: *MFM* Myofibrillar myopathy, GNE UDP-N-acetylglucosamine 2-epimerase/N-acetylmannosamine kinase, *DYSF* dysferlin, *GIPC1* GAIP/RGS19-interacting protein, *LRP12* LDL receptorrelated protein 12, *CRYAB* alphaB-crystallin, *FLNC* filamin C, *DNAJB6* DNAJ/HSP40 homolog, subfamily B, member 6, *TTN* Titin, DM1 Myotonic Dystrophy type 1,BMD Becker muscular dystrophy, *MYH7* Myosin heavy chain 7, *HSPB8* Heat shock protein beta 8, *BAG3* Bcl2-associated athanogene 3, *VCP* Valosin-containing protein, *HNRNPA1* heterogeneous nuclear ribonucleoprotein A1, *ACTN2* alpha-actinin-2, *ACTA*1α-actin 1, *MYOT* myotilin, *ZASP* Z-band alternatively spliced PDZ motif-containing protein, *TIA1* T-cell intracellular antigen-I, *SQSTM1* Sequestosome-1, *MATR3* Matrin-3, *PLIN4* Perilipin 4, *Col6A2* Collagen VI, *TNPO3* Transportin 3, *SMCHD1* Structural maintenance of chromosomes flexible hinge domain containing 1, *HNRNPDL* Heterogeneous nuclear ribonucleoprotein D-like protein, PABPN1 polyadenylate (polyA) binding protein nuclear 1, CAV3 Caveolin-3, CLN3 Lysosomal/Endosomal transmembrane protein, ADSSL1 Adenyl succinate synthase-like 1, FHL1 Four-and-a-half-LIM protein 1, TCAP Telethonin, FKRP Fukutin-related protein, TRIM32 Tripartite-motif containing gene 32, GFPT1 Glutamine: Fructose-6-phosphate Amidotransferase 1, DYS Dystrophin, AR Androgen Receptor, HMERF hereditary myopathy with early respiratory failure, OPDM Oculopharyngodistal Myopathy, ADRVM autosomal dominant rimmed vacuolar myopathy, IBMPFD inclusion body myopathy associated with Paget's disease of bone and frontotemporal dementia, ALS amyotrophic lateral sclerosis, VCPDM vocal cord and pharyngeal weakness with distal myopathy, SBMA spinal and bulbar muscular atrophy, CMT Charcot-Marie-Tooth type, NCL3 Neuronal ceroid lipofuscinosis 3, LGMD Limb-girdle muscular dystrophies, STM sarcotubular myopathy, MRUPAV myopathy with rimmed ubiquitin-positive autophagic vacuolation, OPMD oculopharyngeal muscular dystrophy, FSHD facioscapulohumeral muscular dystrophy, CMS congenital myasthenic syndromes, RV rimmed vacuolar, AL adductor longus, AM adductor magnus, RF rectus femoris, VM vastus medialis, VI vastus intermedius, VL vastus lateralis, G gracilis, S sartorius, ST semitendinosus, SM semimembranosus, BF biceps femoris, TA tibialis anterior, EDL extensor digitorum longus, PL peroneus longus, TP tibialis posterior, SO soleus, GCM gastrocnemius medial, GCL gastrocnemius lateral.

References

1. Bugiardini E, Morrow JM, Shah S, Wood CL, Lynch DS, Pitmann AM, et al. The Diagnostic Value of MRI Pattern Recognition in Distal Myopathies. *Front Neurol* (2018) 9:456. Epub 2018/07/13. doi: 10.3389/fneur.2018.00456.
2. Fischer D, Kley RA, Strach K, Meyer C, Sommer T, Eger K, et al. Distinct muscle imaging patterns in myofibrillar myopathies. *Neurology* (2008) 71(10):758-65.doi: 10.1212/01.wnl.0000324927.28817.9b.
3. Bortolani S, Fattori F, Monforte M, Ricci E, Tasca G. Peculiar muscle imaging findings in a patient with alphaB-crystallinopathy and axial myopathy. *Journal of the neurological sciences* (2020) 416:116999. doi: 10.1016/j.jns.2020.116999.
4. Olivé M, Odgerel Z, Martínez A, Poza JJ, Bragado FG, Zabalza RJ, et al. Clinical and myopathological evaluation of early- and late-onset subtypes of myofibrillar myopathy. *Neuromuscul Disord* (2011) 21(8):533-42. doi: 10.1016/j.nmd.2011.05.002.
5. Kley RA, Serdaroglu-Oflazer P, Leber Y, Odgerel Z, van der Ven PF, Olivé M, et al. Pathophysiology of protein aggregation and extended phenotyping in filaminopathy. *Brain* (2012) 135(Pt 9):2642-60. doi: 10.1093/brain/aws200.
6. Schramm N, Born C, Weckbach S, Reilich P, Walter MC, Reiser MF. Involvement patterns in myotilinopathy and desminopathy detected by a novel neuromuscular whole-body MRI protocol. *Eur Radiol* (2008) 18(12):2922-36. doi: 10.1007/s00330-008-1071-1.
7. Hamaguchi M, Kokubun N, Inoue M, Komagamine T, Aoki R, Nishino I, et al. A family with adult-onset myofibrillar myopathy with BAG3 mutation (P470S) presenting with axonal polyneuropathy. *Neuromuscul Disord* (2020) 30(9):727-31. doi: 10.1016/j.nmd.2020.07.012.
8. Liu CY, Yao J, Kovacs WC, Shrader JA, Joe G, Ouwerkerk R, et al. Skeletal Muscle Magnetic Resonance Biomarkers in GNE Myopathy. *Neurology* (2021) 96(5):e798-e808. doi: 10.1212/wnl.0000000000011231.
9. Mahjneh I, Lamminen AE, Udd B, Paetau AE, Hackman P, Korhola OA, et al. Muscle magnetic resonance imaging shows distinct diagnostic patterns in Welander and tibial muscular dystrophy. *Acta Neurol Scand* (2004) 110(2):87-93. doi: 10.1111/j.1600-0404.2004.00283.x.
10. Bucelli RC, Arhzaouy K, Pestronk A, Pittman SK, Rojas L, Sue CM, et al. SQSTM1 splice site mutation in distal myopathy with rimmed vacuoles. *Neurology* (2015) 85(8):665-74. doi: 10.1212/wnl.0000000000001864.
11. Niu Z, Pontifex CS, Berini S, Hamilton LE, Naddaf E, Wieben E, et al. Myopathy With SQSTM1 and TIA1 Variants: Clinical and Pathological Features. *Front Neurol* (2018) 9:147. doi: 10.3389/fneur.2018.00147.
12. Evilä A, Vihola A, Sarparanta J, Raheem O, Palmio J, Sandell S, et al. Atypical phenotypes in titinopathies explained by second titin mutations. *Ann Neurol* (2014) 75(2):230-40. doi: 10.1002/ana.24102.
13. Palmio J, Leonard-Louis S, Sacconi S, Savarese M, Penttila S, Semmler AL, et al. Expanding the importance of HMERF titinopathy: new mutations and clinical aspects. *J Neurol* (2019) 266(3):680-90. doi: 10.1007/s00415-019-09187-2.
14. Diaz-Manera J, Fernandez-Torron R, J LL, James MK, Mayhew A, Smith FE, et al. Muscle MRI in patients with dysferlinopathy: pattern recognition and implications for clinical trials. *Journal of neurology, neurosurgery, and psychiatry* (2018) 89(10):1071-81. doi: 10.1136/jnnp-2017-317488.
15. Fiorillo C, Astrea G, Savarese M, Cassandrini D, Brisca G, Trucco F, et al. MYH7-related

myopathies: clinical, histopathological and imaging findings in a cohort of Italian patients. *Orphanet J Rare Dis* (2016) 11(1):91. doi: 10.1186/s13023-016-0476-1.
16. Zhao J, Liu J, Xiao J, Du J, Que C, Shi X, et al. Clinical and muscle imaging findings in 14 mainland chinese patients with oculopharyngodistal myopathy. *PLoS One* (2015) 10(6):e0128629. doi: 10.1371/journal.pone.0128629.
17. Kumutpongpanich T, Ogasawara M, Ozaki A, Ishiura H, Tsuji S, Minami N, et al. Clinicopathologic Features of Oculopharyngodistal Myopathy With LRP12 CGG Repeat Expansions Compared With Other Oculopharyngodistal Myopathy Subtypes. *JAMA Neurol* (2021). doi: 10.1001/jamaneurol.2021.1509.
18. Ghaoui R, Palmio J, Brewer J, Lek M, Needham M, Evilä A, et al. Mutations in HSPB8 causing a new phenotype of distal myopathy and motor neuropathy. *Neurology* (2016) 86(4):391-8. doi: 10.1212/wnl.0000000000002324.
19. Al-Tahan S, Weiss L, Yu H, Tang S, Saporta M, Vihola A, et al. New family with HSPB8-associated autosomal dominant rimmed vacuolar myopathy. *Neurol Genet* (2019) 5(4):e349. doi: 10.1212/nxg.0000000000000349.
20. Guo X, Zhao Z, Shen H, Qi B, Li N, Hu J. VCP myopathy: A family with unusual clinical manifestations. *Muscle & nerve* (2019) 59(3):365-9. doi: 10.1002/mus.26389.
21. Palmio J, Sandell S, Suominen T, Penttilä S, Raheem O, Hackman P, et al. Distinct distal myopathy phenotype caused by VCP gene mutation in a Finnish family. *Neuromuscul Disord* (2011) 21(8):551-5. doi: 10.1016/j.nmd.2011.05.008.
22. Hackman P, Rusanen SM, Johari M, Vihola A, Jonson PH, Sarparanta J, et al. Dominant Distal Myopathy 3 (MPD3) Caused by a Deletion in the HNRNPA1 Gene. *Neurol Genet* (2021) 7(6):e632. doi: 10.1212/nxg.0000000000000632.
23. Mensch A, Kraya T, Koester F, Muller T, Stoevesandt D, Zierz S. Whole-body muscle MRI of patients with MATR3-associated distal myopathy reveals a distinct pattern of muscular involvement and highlights the value of whole-body examination. *J Neurol* (2020) 267(8):2408-20. doi: 10.1007/s00415-020-09862-9.
24. Ban R, Zhang Y, Li K, Shi Q. A Case of Myotonic Dystrophy Type I With Rimmed Vacuoles in Skeletal Muscle Pathology. *J Clin Rheumatol* (2020). doi: 10.1097/RHU.0000000000001496.
25. Chen S, Huang P, Qiu Y, Zhou Q, Li X, Zhu M, et al. Phenotype variability and histopathological findings in patients with a novel DNM2 mutation. *Neuropathology* (2018) 38(1):34-40. doi: 10.1111/neup.12432.
26. Radke J, Koll R, Gill E, Wiese L, Schulz A, Kohlschütter A, et al. Autophagic vacuolar myopathy is a common feature of CLN3 disease. *Ann Clin Transl Neurol* (2018) 5(11):1385-93. doi: 10.1002/acn3.662.
27. Kojima Y, Noto YI, Takewaki D, Tokuda N, Shiga K, Hamano A, et al. Characteristic Posterior-dominant Lower Limb Muscle Involvement in Limb-girdle Muscular Dystrophy due to a DNAJB6 Phe93Leu Mutation. *Intern Med* (2017) 56(17):2347-51. doi: 10.2169/internalmedicine.6957-15.
28. Kim K, Park HJ, Lee JH, Hong J, Ahn SW, Choi YC. Two Korean Families with Limb-Girdle Muscular Dystrophy Type 1D Associated with DNAJB6 Mutations. *Yonsei Med J* (2018) 59(5):698-701. doi: 10.3349/ymj.2018.59.5.698.
29. Vihola A, Palmio J, Danielsson O, Penttilä S, Louiselle D, Pittman S, et al. Novel mutation in TNPO3 causes congenital limb-girdle myopathy with slow progression. *Neurol Genet* (2019) 5(3):e337. doi: 10.1212/nxg.0000000000000337.

30. Sun Y, Chen H, Lu Y, Duo J, Lei L, OuYang Y, et al. Limb girdle muscular dystrophy D3 HNRNPDL related in a Chinese family with distal muscle weakness caused by a mutation in the prion-like domain. *J Neurol* (2019) 266(2):498-506. doi: 10.1007/s00415-018-9165-4.
31. Berardo A, Lornage X, Johari M, Evangelista T, Cejas C, Barroso F, et al. HNRNPDL-related muscular dystrophy: expanding the clinical, morphological and MRI phenotypes. *J Neurol* (2019) 266(10):2524-34. doi: 10.1007/s00415-019-09437-3.
32. Chamova T, Bichev S, Todorov T, Gospodinova M, Taneva A, Kastreva K, et al. Limb girdle muscular dystrophy 2G in a religious minority of Bulgarian Muslims homozygous for the c.75G>A, p.Trp25X mutation. *Neuromuscul Disord* (2018) 28(8):625-32. doi: 10.1016/j.nmd.2018.05.005.
33. Chen H, Xu G, Lin F, Jin M, Cai N, Qiu L, et al. Clinical and genetic characterization of limb girdle muscular dystrophy R7 telethonin-related patients from three unrelated Chinese families. *Neuromuscul Disord* (2020) 30(2):137-43. doi: 10.1016/j.nmd.2019.12.004.
34. Leung DG, Bocchieri AE, Ahlawat S, Jacobs MA, Parekh VS, Braverman V, et al. Longitudinal functional and imaging outcome measures in FKRP limb-girdle muscular dystrophy. *BMC Neurol* (2020) 20(1):196. doi: 10.1186/s12883-020-01774-5.
35. Wei XJ, Miao J, Kang ZX, Gao YL, Wang ZY, Yu XF. A novel homozygous exon2 deletion of TRIM32 gene in a Chinese patient with sarcotubular myopathy: A case report and literature review. *Bosn J Basic Med Sci* (2021) 21(4):495-500. doi: 10.17305/bjbms.2020.5288.
36. Yang K, Zeng YH, Qiu YS, Lin F, Chen HZ, Jin M, et al. Expanding the phenotype and genotype spectra of PLIN4-associated myopathy with rimmed ubiquitin-positive autophagic vacuolation. *Acta Neuropathol* (2022) 143(6):733-5. doi: 10.1007/s00401-022-02422-7.
37. Alonso-Jimenez A, Kroon R, Alejaldre-Monforte A, Nunez-Peralta C, Horlings CGC, van Engelen BGM, et al. Muscle MRI in a large cohort of patients with oculopharyngeal muscular dystrophy. *Journal of neurology, neurosurgery, and psychiatry* (2019) 90(5):576-85. doi: 10.1136/jnnp-2018-319578.
38. Holm-Yildiz S, Witting N, Dahlqvist J, de Stricker Borch J, Solheim T, Fornander F, et al. Permanent muscle weakness in hypokalemic periodic paralysis. *Neurology* (2020) 95(4):e342-e52. doi: 10.1212/wnl.0000000000009828.
39. Jia BX, Yang Q, Li SY, Wan M, Wang H, Huo LY, et al. Muscle edema of the lower limb determined by MRI in Asian hypokalaemic periodic paralysis patients. *Neurol Res* (2015) 37(3):246-52. doi: 10.1179/1743132814y.0000000440.
40. Evilä A, Arumilli M, Udd B, Hackman P. Targeted next-generation sequencing assay for detection of mutations in primary myopathies. *Neuromuscul Disord* (2016) 26(1):7-15. doi: 10.1016/j.nmd.2015.10.003.
41. Schreckenbach T, Henn W, Kress W, Roos A, Maschke M, Feiden W, et al. Novel FHL1 mutation in a family with reducing body myopathy. *Muscle & nerve* (2013) 47(1):127-34. doi: 10.1002/mus.23500.
42. Finlayson S, Morrow JM, Rodriguez Cruz PM, Sinclair CD, Fischmann A, Thornton JS, et al. Muscle magnetic resonance imaging in congenital myasthenic syndromes. *Muscle & nerve* (2016) 54(2):211-9. doi: 10.1002/mus.25035.
43. Zhong J, Xie Y, Dang Y, Zhang J, Song Y, Lan D. Use of RNA‑sequencing to detect abnormal transcription of the collagen α‑2 (VI) chain gene that can lead to Bethlem myopathy. *International journal of molecular medicine* (2021) 47(3). doi: 10.3892/ijmm.2021.4861.
44. Kan HE, Klomp DW, Wohlgemuth M, van Loosbroek-Wagemans I, van Engelen BG, Padberg

GW, et al. Only fat infiltrated muscles in resting lower leg of FSHD patients show disturbed energy metabolism. *NMR Biomed* (2010) 23(6):563-8. doi: 10.1002/nbm.1494.
45. Oatmen K, Camelo-Piragua S, Zaghloul N. Novel mutation in the MYH2 gene in a symptomatic neonate with a hereditary myosin myopathy. *J Neonatal Perinatal Med* (2022) 15(1):63-8. doi: 10.3233/npm-210780.
46. Castiglioni C, Cassandrini D, Fattori F, Bellacchio E, D'Amico A, Alvarez K, et al. Muscle magnetic resonance imaging and histopathology in ACTA1-related congenital nemaline myopathy. *Muscle & nerve* (2014) 50(6):1011-6. doi: 10.1002/mus.24353.
47. Ishiguro K, Nakayama T, Yoshioka M, Murakami T, Kajino S, Shichiji M, et al. Characteristic findings of skeletal muscle MRI in caveolinopathies. *Neuromuscul Disord* (2018) 28(10):857-62. doi: 10.1016/j.nmd.2018.07.010.
48. Khosa S, Shu FY, Khanlou N, Mishra SK. A Case of Becker Muscular Dystrophy With Rimmed Vacuoles and Normal Dystrophin. *J Clin Rheumatol* (2020) 26(8):e307-e8. doi: 10.1097/RHU.0000000000001161.
49. Klickovic U, Zampedri L, Sinclair CDJ, Wastling SJ, Trimmel K, Howard RS, et al. Skeletal muscle MRI differentiates SBMA and ALS and correlates with disease severity. *Neurology* (2019) 93(9):e895-e907. doi: 10.1212/wnl.0000000000008009.
